# Ablation of glucocorticoid receptor in the hindbrain of the mouse provides a novel model to investigate stress disorders

**DOI:** 10.1038/s41598-019-39867-y

**Published:** 2019-03-01

**Authors:** Anne-Louise Gannon, Laura O’Hara, J. Ian Mason, Diane Rebourcet, Sarah Smith, Adriana Traveres, Carlos Jose Alcaide-Corral, Hanne Frederiksen, Anne Jørgensen, Laura Milne, Rod T. Mitchell, Lee B. Smith

**Affiliations:** 1 MRC Centre for Reproductive Health, University of Edinburgh, The Queen’s Medical Research Institute, 47 Little France Crescent, Edinburgh, EH16 4TJ UK; 2 Centre for Discovery Brain Sciences, Hugh Robson Building, George Square, Edinburgh, EH8 9XD UK; 3 Edinburgh Preclinical Imaging, College of Medicine and Veterinary Medicine, University of Edinburgh, Edinburgh, EH16 4TJ UK; 4 Department of Growth and Reproduction, Rigshospitalet, University of Copenhagen, Copenhagen, Denmark; 5 International Centre for Research and Research Training in Endocrine Disruption of Male Reproduction and Child Health (EDMaRC), Rigshospitalet, Denmark; 6 School of Environmental and Life Sciences, Faculty of Science, University of Newcastle, Callaghan, 2308 NSW Australia

## Abstract

The hypothalamic-pituitary-adrenal (HPA) axis regulates responses to internal and external stressors. Many patients diagnosed with conditions such as depression or anxiety also have hyperactivity of the HPA axis. Hyper-stimulation of the HPA axis results in sustained elevated levels of glucocorticoids which impair neuronal function and can ultimately result in a psychiatric disorder. Studies investigating Glucocorticoid Receptor (GR/NR3C1) in the brain have primarily focused on the forebrain, however in recent years, the hindbrain has become a region of interest for research into the development of anxiety and depression, though the role of GR signalling in the hindbrain remains poorly characterised. To determine the role of glucocorticoid signalling in the hindbrain we have developed a novel mouse model that specifically ablates hindbrain GR to ascertain its role in behaviour, HPA-axis regulation and adrenal structure. Our study highlights that ablation of GR in the hindbrain results in excessive barbering, obsessive compulsive digging and lack of cage exploration. These mice also develop kyphosis, elevated circulating corticosterone and severe adrenal cortex disruption. Together, this data demonstrates a role for hindbrain GR signalling in regulating stress-related behaviour and identifies a novel mouse model to allow further investigation into the pathways impacting stress and anxiety.

## Introduction

According to the World Health Organization, mood disorders will be the second leading cause of disability by the year 2020,  hence the need for appropriate models to understand these disorders is essential. Intense or chronic bouts of stress have been shown to result in emotional disturbances and hormonal disruption that can ultimately culminate in a mood disorder^1^. In patients that have been diagnosed with conditions such as depression or anxiety, more than 50% of these have hyperactivity of the Hypothalamic-pituitary-adrenal (HPA) axis^2,3^. HPA-axis activation results in low affinity for food, decreased sex drive, increased blood flow to muscle, increased locomotive activity and raised blood glucose which prime the body to respond to a stress event^4^. In normal conditions these bursts of activity last for only a few minutes at a time, however, prolonged stress is thought to overstimulate the HPA axis causing hypersecretion of cortisol and can ultimately, if left untreated, lead to the dysregulation of the HPA axis, potentially promoting the onset of a mood disorder^5^.

Cortisol is known to be important in regulating neuronal survival, neuron excitability, neurogenesis, and memory acquisition^6^. It is thought that prolonged periods of increased cortisol can impair these functions. On a molecular level, cortisol primarily exerts its effects through the glucocorticoid receptor (GR), a member of the nuclear receptor transcription factor superfamily *(Nr3c1)*. Previous studies have highlighted the role of GR in the brain and its control of stress and cognitive function. The current challenge facing this field is the two relevant but opposing concepts. The detrimental effects of excessive glucocorticoid levels on the hippocampus seems to require GR to be functioning normally, whereas these same excessive glucocorticoid levels in conditions such as depression, may result from impaired negative feedback inhibition on the HPA axis, which is caused by loss of function of GR^4^. For these reasons, more research is needed into the various regions of the brain expressing GR and their role in depression and anxiety which can be achieved through the development of novel models. The initial body of research investigating mood disorders focused upon fast acting neurotransmitters such as serotonin, norepinephrine and dopamine depletion. Despite this, these neurotransmitter systems are short term and cannot account for the progressive increase in disease severity observed over time in patients with anxiety or depression, therefore, research has expanded into brain regions out-with the forebrain and cell signalling pathways that could account for the pathology observed overtime in patients^7^. The hindbrain, known for its role in autonomous regulation, has in recent years become a region of interest for research into the development of anxiety and depression. Studies conducted by Zhang *et al*. demonstrated that glucocorticoids are important for tuning hindbrain stress integration via GR expression in the nucleus of the solitary tract (NTS)^6^. Studies have also demonstrated increased glucocorticoids impacting neuronal plasticity^4^.

Using mouse models to mimic any human behaviour is an extremely difficult undertaking, especially in relation to mood disorders which are often multifactorial. The signs and symptoms exhibited by patients are often heterogeneous^8^. For example, some patients experience excessive weight gain while others with the same condition experience weight loss or chronic fatigue and insomnia^9^. In addition to this, a mouse’s response to stress differs due to the lack of conscious self^10^. However, stress behaviours that can be measured and quantified have been identified in rodents permitting investigation of endocrinological and behavioural alterations to better understand the aetiology of mood disorders. To support this, we have generated a novel mouse model that ablates GR in the hindbrain, to investigate its impact on behaviour and the HPA axis. We demonstrate that ablation of GR in the hindbrain results in a stress-sensitive phenotype in mice, with excessive barbering, obsessive compulsive digging and lack of cage exploration. These mice also develop kyphosis, elevated circulating corticosterone and severe adrenal cortex disruption, suggesting that this novel mouse model represents a suitable experimental system with which to investigate mood disorders.

## Results

### Confirmation of GR recombination in the hindbrain

**Figure 1:**
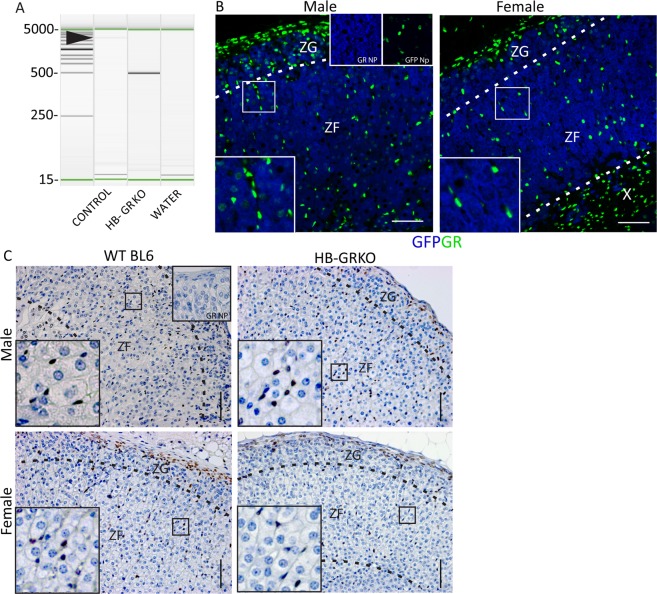
Conformation of recombination of GR in the Hindbrain. (**A**) PCR interrogation of genomic PCR confirms recombination of floxed GR in the hindbrain, with WT GR at 2.5 kb and recombined GR at 500 bp. (**B**) Immunohistochemical localisation of GFP and GR in the adrenal of *Cyp11a1*^+/GC^: R26-EYFP mice show that despite the Cre expression in the adrenal cortex, it does not target GR positive cells. (**C**) Immunohistochemical localisation of GR in the adrenal cortex of HB-GRKO shows that GR has not been targeted in the adrenal cortex and show no differences in localisation when compared with WT BL6 mice. Insert denoted by squares. Abbreviations; ZG = zona glomerulosa, ZF = zona fasciculata. Np = No primary. Scale bars 100 µm.

We previously generated a mouse model that is able to ablate target genes from steroidogenic cell types through the use of a GFP-Cre-GC targeted to the mouse *Cyp11a1* locus to drive Cre Recombinase expression^11^. Using this Cre, we are able to ablate GR from the hindbrain, evidenced through interrogation of hindbrain genomic DNA (Fig. 1A). As the Cre expression is driven by a *Cyp11a1* promoter, and *Cyp11a1* is widely expressed throughout the adrenal cortex, we interrogated expression of the transgene in the adrenal gland in order to fully understand the sites of targeting that could underpin any observable phenotype. Through use of *Cyp11a1*^+/GC^: R26-EYFP mice and double immunofluorescence localisation we are able to rule out this potential confounder since the analysis shows that the Cre does not target GR expressing cell types in the adrenal (Fig. 1B). GR-expressing cells are located between the Cre-expressing cells confirming that the *Cyp11a1-Cre* is unable to target these cells (Fig. 1C). Immunohistochemistry analysis confirms this observation, with no GR ablation visible in the adrenal cortex of hindbrain-glucocorticoid receptor knockout (HB-GRKO) mice (Fig. 1D). This Cre is also expressed in Leydig cells in the testis, however targeting efficiency is <20% and this has previously been shown to have no impact on testis function, circulating testosterone or Luteinizing hormone. These results demonstrate that any adrenal phenotype arising in these mice results from ablating GR outside of the adrenal.

### HB-GRKO mice show stressed behaviours via excessive barbering and digging

**Figure 2:**
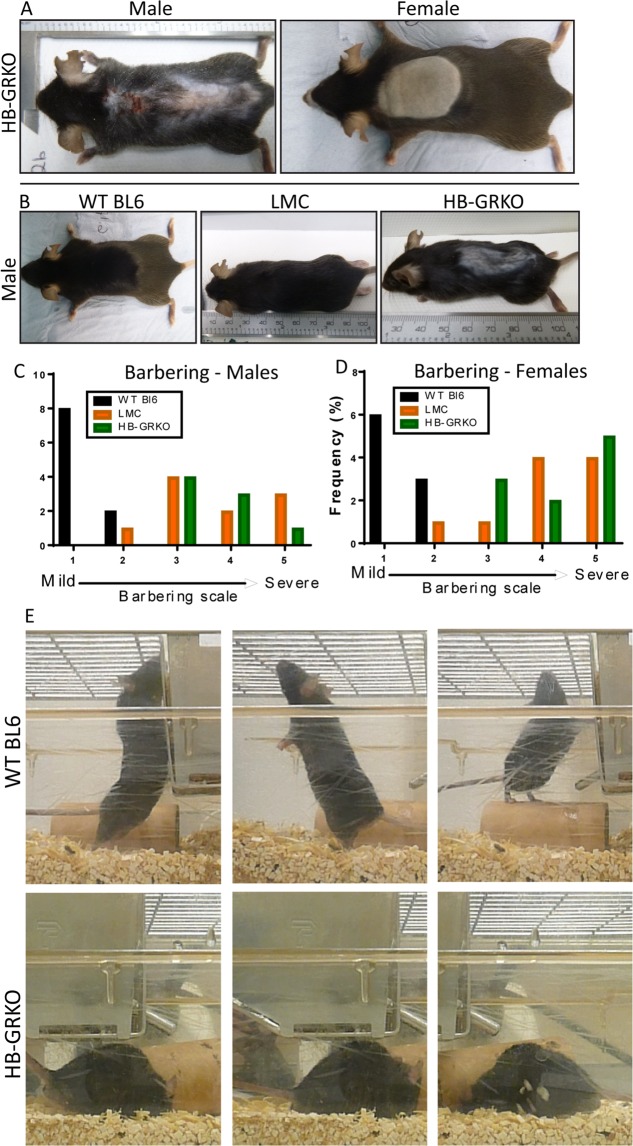
HB-GRKO mice display severe barbering and cage stress. (**A**) Whole body examination shows excessive barbering to face and back in male and females, often resulting in broken skin and complete removal of whiskers. (**B**) Individually caged WT BL6 and LMC males displaying no hair loss or barbering. Individually caged HB-GRKO male shows excessive barbering and broken skin. (**C**) Histograph detailing distribution of genotypes and barbering severity in males, showing HB-GRKO mice with a barbering score of 3–5, compared to WT BL6 mice that fell between 0–1 on the scale. (**D**) Histograph detailing distribution of genotypes and barbering severity in females, showing HB-GRKO mice with a barbering score of 3–5, compared to WT BL6 mice that fell between 0–1 on the scale. (**E**) Observations of cage behaviour over a 5 minute period. WT BL6 animals can be seen exploring that cage with multiple rearing observed. HB-GRKO mice did little cage exploration and display obsessive behaviour via excessive and compulsive digging.

As disruption to GR is known to play a role in stress and anxiety^2,4,12^, 40 cages consisting of 4–6 male or female mice of mixed genotypes including Cre negative heterozygous littermate controls (Het LMC), Cre negative homozygous littermate controls (LMC), Cre positive heterozygous hindbrain glucocorticoid receptor knockouts (Het HB-GRKO) and Cre positive homozygous hindbrain glucocorticoid receptor knockouts (HB-GRKO), were first observed for signs of behaviour consistent with stress. Due to the known ability for social stressors on littermate controls^13^ potentially acting as a confounder, an external C57BL6 cohort (WT BL6) were also included in the analysis as an additional control. These mice were bred and held in the same rack as the HB-GRKO colony but had no direct contact with HB-GRKO mice. Barbering in mice is a normal social behaviour, yet, excessive or abnormal barbering has been associated with stress and anxiety^14,15^. Both male and female HB-GRKO animals display excessive barbering resulting in hair loss from large portions of the face, back and stomach (Fig. 2A). Hair loss can also be seen in LMCs. Upon observation, HB-GRKO mice were seen barbering LMCs, and this suggested that HB-GRKO mice were the cause of the excess cage barbering. In support of this, individually housed WT BL6 mice and LMCs showed no hair removal, however, individually housed HB-GRKO mice self-barber, frequently resulting in excessive hair removal, occasionally severe enough to break the skin (Fig. 2B). A barbering scale was generated to determine severity of the hair loss between the genotypes in this study^14,15^. According to our scale, HB-GRKO mice had a barbering score of between 3 and 5, which is above the accepted limit for normal husbandry barbering. Furthermore, cages containing a higher number of HB-GRKO animals demonstrated some of the most severe barbering, all falling within 4–5 on our barbering scale. This is in stark contrast to WT BL6 mice that had a barbering score between 0–1 (Fig. 2C,D). Monitoring of cage behaviour prior to collection revealed that WT BL6 mice explored the cage, with multiple rearing attempts made (normal behaviour associated with the explorative nature of mice)^16^. In contrast, HB-GRKO mice spent little time exploring and focused on repetitive digging in the same spot, behaviour characteristic of a stressed state (Fig. 2D). Interestingly, stress behaviour and severe hair loss through barbering does not present until at least day (d)90. Cre expression in the hindbrain is not observed in embryonic development but becomes expressed postnatally. This could account for the late development of the stress-like phenotype. In addition to barbering, general cage behaviour was also observed over a five minute period. WT BL6 mice had an average of 14 rears over this time period, compared to only 3 in HB-GRKO mice (Fig. 2D). Together this data suggests that loss of GR signalling in the hindbrain results in increased stress-related behaviour. Having established this, we next wanted to ascertain whether these behavioural changes impacted overall body condition and weight.

### HB-GRKO mice show alterations in body weight and spinal curvature

**Figure 3:**
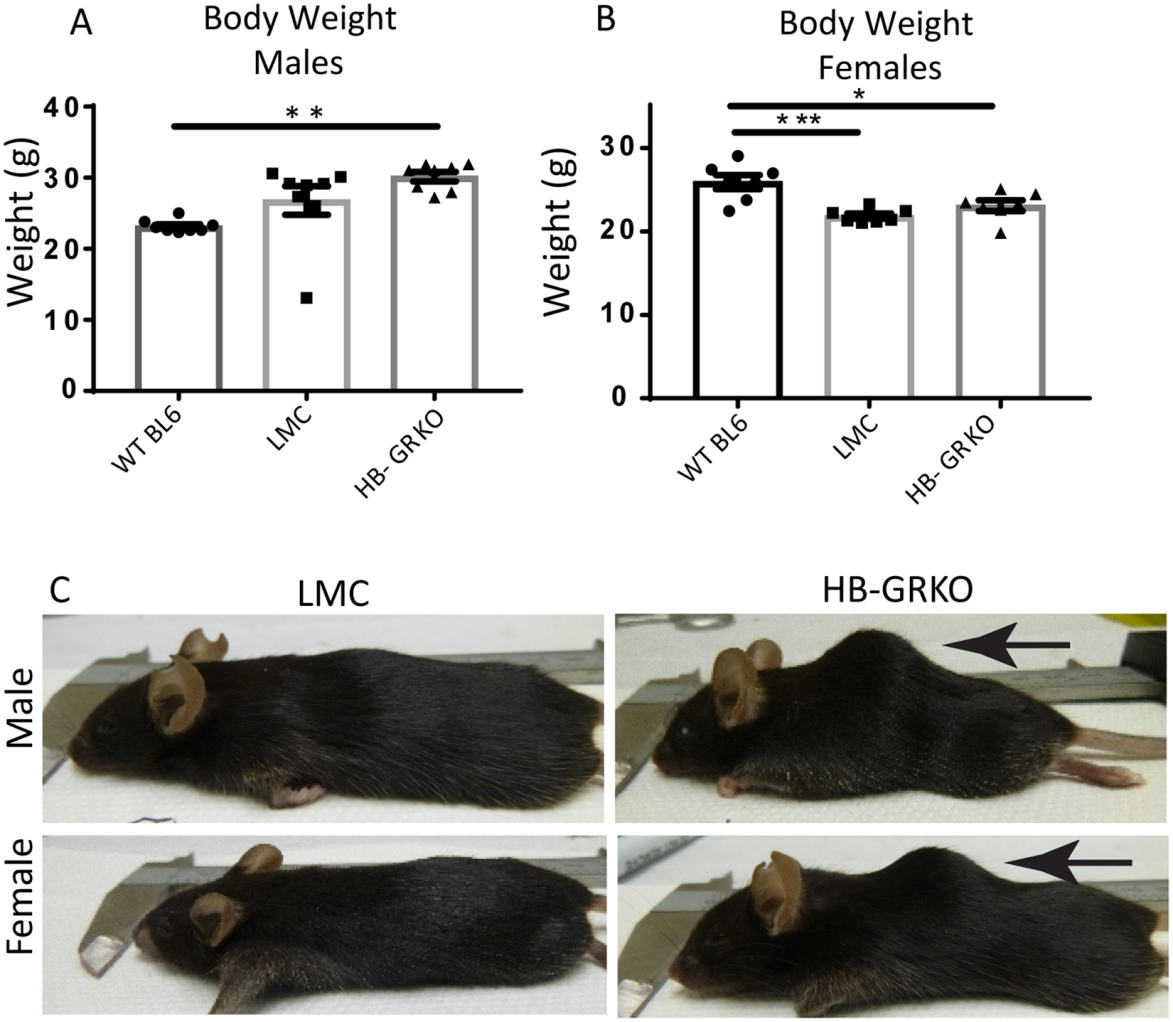
HB-GRKO mice show alterations in body weight and spinal curvature. (**A**) Male body weights compared to an external WT BL6 cohort reveals a significant increase in HB-GRKO mice (one-way ANOVA; n = 7–8, **p < 0.001, error bars SEM). There is no difference in body weight of HB-GRKO LMC compared to WT BL6. (**B**) Female body weights compared to WT BL6 show a significant decrease in in both in HB-GRKO LMC and HB-GRKO mice (one-way ANOVA; n = 8, ***p < 0.0001, *p < 0.05 error bars SEM). (**C**) Whole body images showing exaggerated spinal curvature in HB-GRKO male and females. Spinal curvature denoted by arrows.

Body weight is significantly increased in HB-GRKO males compared to male WT BL6 mice (Fig. 3A), whilst in contrast, a significant decrease in body weight is observed in female LMCs and HB-GRKO mice compared to female WT BL6 mice (Fig. 3B). This decrease in weight is also observed in Het LMC and Het HB-GRKO females (Sup Fig. 2B). During collection it could be seen that HB-GRKO mice had excessive spinal curvature in both males and females (Fig. 3C). For these reasons, PET/CT analysis was used to investigate the spinal curvature in HB-GRKO mice.

### Positron emission tomography-computed tomography PET/CT imaging confirms kyphosis in HB-GRKO mice

**Figure 4:**
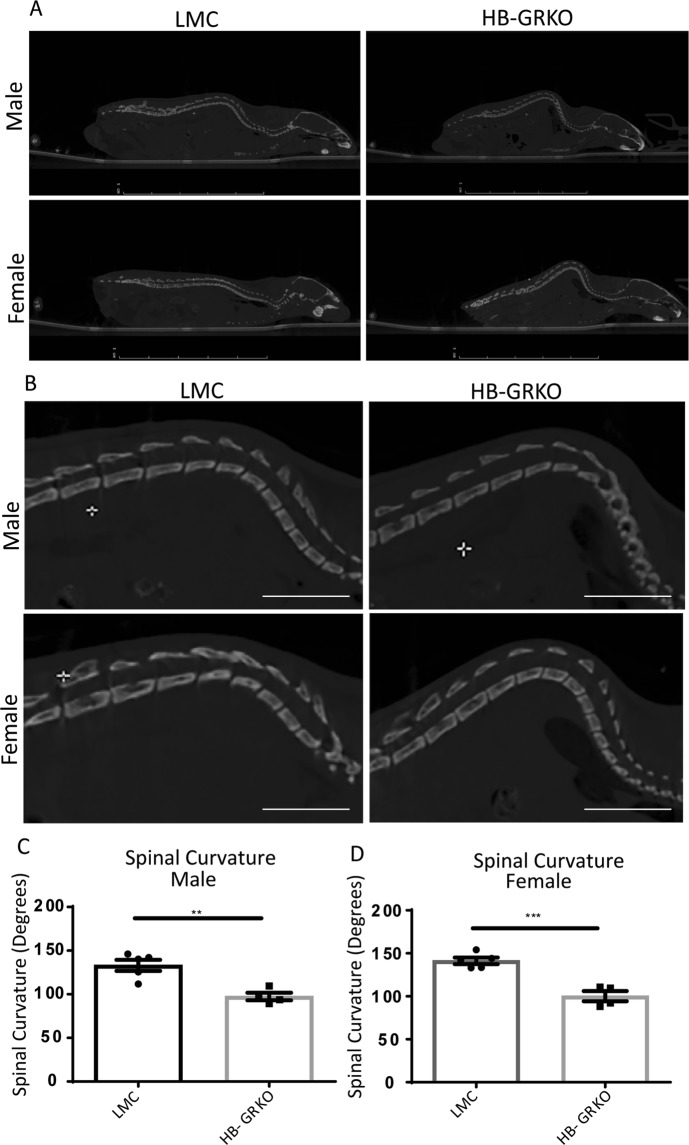
Excessive spinal curvature confirmed through PET/CT analysis. (**A**) CT scans show that in HB-GRKO mice, there was an increase in spinal curvature and show spinal collapse. (**B**) Closer investigation of the spine shows no fusing of vertebrae in HB-GRKO. (**C**,**D**) Interrogation of the anterior angle of the spine shows a significant decrease in HB-GRKO mice when compared to littermate controls. (Unpaired Student T-test;**p < 0.001, ***p < 0.0001. error bars SEM). Scale bars 1 cm.

Micro-CT scans were taken to permit analysis of spinal curvature and bone condition. WT BL6 mice were not included in this part of the analysis as LMCs did not show increased spinal curvature from initial observations. These results revealed an exaggerated spinal curvature in HB-GRKO male and female mice compared to LMC males and females (Fig. 4A). Closer inspection of the vertebrae was used to establish whether this curvature is a result of vertebral fusing, however, results show no fusing of the vertebrae in any group analysed (Fig. 4B). The absence of vertebral fusing in these mice suggests that the problem is not developmental in nature. As per standard orthopaedic practice, the inward angle of the spine was measured to quantify the extent of the curvature observed in HB-GRKO mice^17^. The inward angle of the spine in both HB-GRKO males and females decreased from 140° to 100° when compared to LMCs, demonstrating the development of kyphosis (Fig. 4C,D). This increase curvature was also observed in Het LMC and Het HB-GRKO male and females (Sup Fig. 2D,E). The observation of stress behaviours and increased spinal curvature suggested that the adrenal gland was also been impacted, so analysis of adrenal weight, morphology, stress and functional cortex markers was performed.

### HB-GRKO leads to severe adrenal cortex disruption

**Figure 5:**
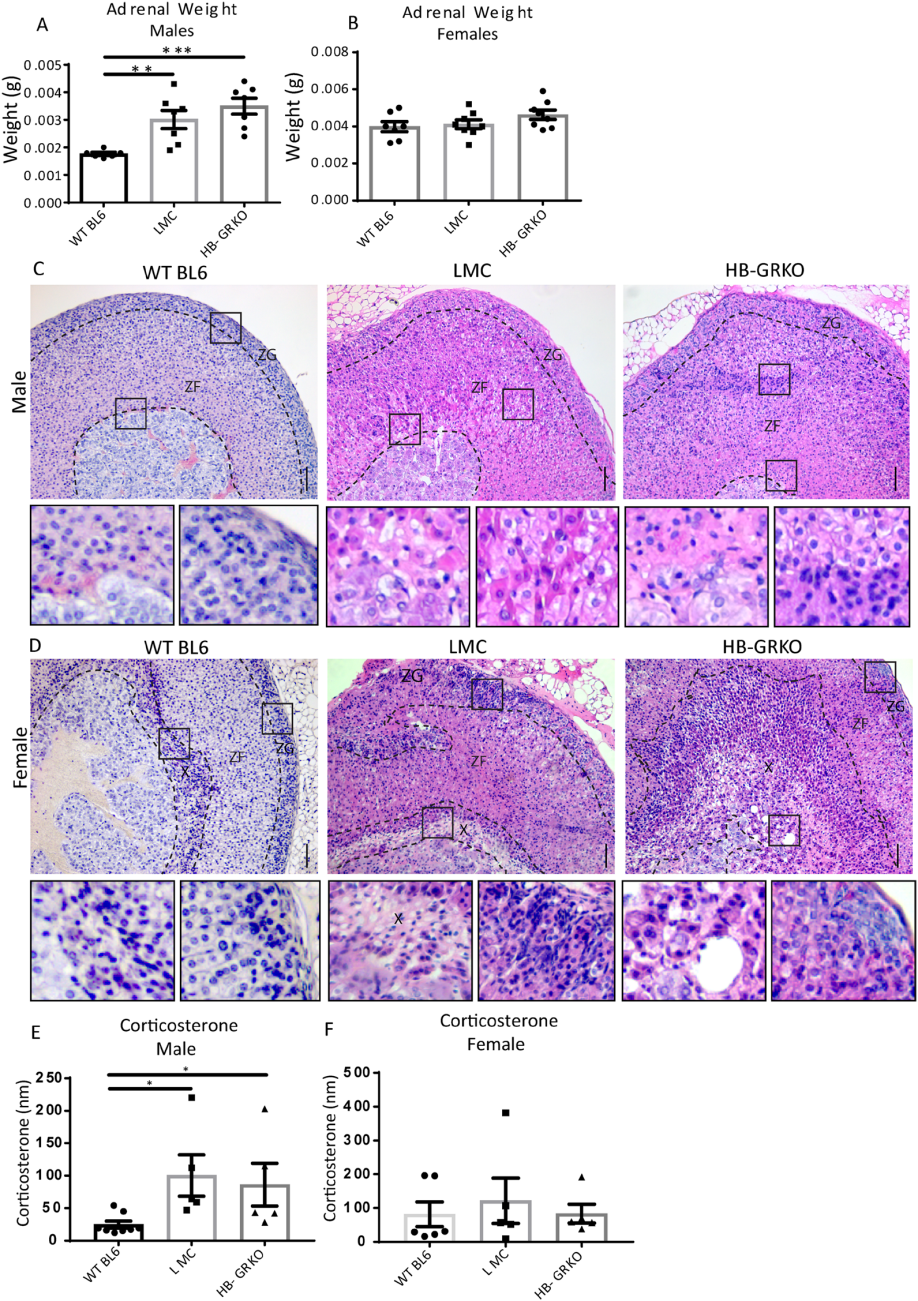
HB-GRKO LMC and HB-GRKO present with severely disrupted adrenals. (**A**) Analysis of male adrenal weight revealed an increase in weight in both HB-GRKO LMC and HB-GRKO mice compared to BL6 controls (One-way ANOVA; n = 8, ***p < 0.0001, **p < 0.001, error bars SEM). (**B**) Analysis of female adrenal weight revealed no differences between any of the cohorts and WT BL6 controls. (**C**) Morphology analysis of male HB-GRKO LMC and HB-GRKO adrenals both displayed major disruption to the entire cortex. Due to the disruption observed in littermate controls, an external WT BL6 cohort was included in the analysis. Enlarged images of regions of interest denoted by squares. (**D**) Morphology analysis of female HB-GRKO LMC and HB-GRKO adrenals both displayed major disruption to the entire cortex, with abnormal X-zone morphology. Enlarged images of regions of interest denoted by squares. (**E**) Circulating corticosterone levels are elevated in both HB-GRKO LMC and HB-GRKO males compared to WT BL6 (one-way ANOVA; n = 8, *p < 0.05, *p < 0.05, error bars SEM). (**F**) Circulating corticosterone levels do not change in HB-GRKO LMC and HB-GRKO females compared to BL6 controls. Abbreviations; ZG = zona glomerulosa, ZF = zona fasciculata, X = X-zone. Scale bars 100 µm.

Interrogation of adrenal weights revealed a significant increase in HB-GRKO and LMC male mice compared to WT BL6 males (Fig. 5A). No changes in adrenal weight were observed in females (Fig. 5B). Additionally, to rule out that the disruption observed in LMCs was a result of the floxed GR, GR floxed male adrenals were also examined, adrenal morphology was normal in these animals (Sup Fig. 1A). Histological analysis of the adrenal glands in male HB-GRKO mice reveals severe disruption to the cortex with disorganised cortical zones. Examination of male LMCs reveal a similar level of disruption to their adrenal cortex. However male WT BL6 mice have no such disruption to the adrenal, suggesting that the impact on LMC adrenals is also likely related to their being housed with HB-GRKO mice (Fig. 5C). The adrenals of female HB-GRKO and LMCs mice show a similar disruption to the cortex as seen in the males, with the addition of enlargement of the X-zone, which occupied a large portion of the cortex compared to the X-zone in female WT BL6 mice (Fig. 5D). These results are again recapitulated in Het LMC and Het HB-GRKO mice (Sup Fig. 3C). Analysis of circulating serum corticosterone revealed significantly increased levels in HB-GRKO and LMC males compared to WT BL6 mice (Fig. 5E), surprisingly, this increase was not observed in females of any genotype. Elevated levels of serum corticosterone could explain the development of kyphosis in these mice, as it has been noted that patients with Cushing’s Syndrome present with kyphosis^18^.

**Figure 6:**
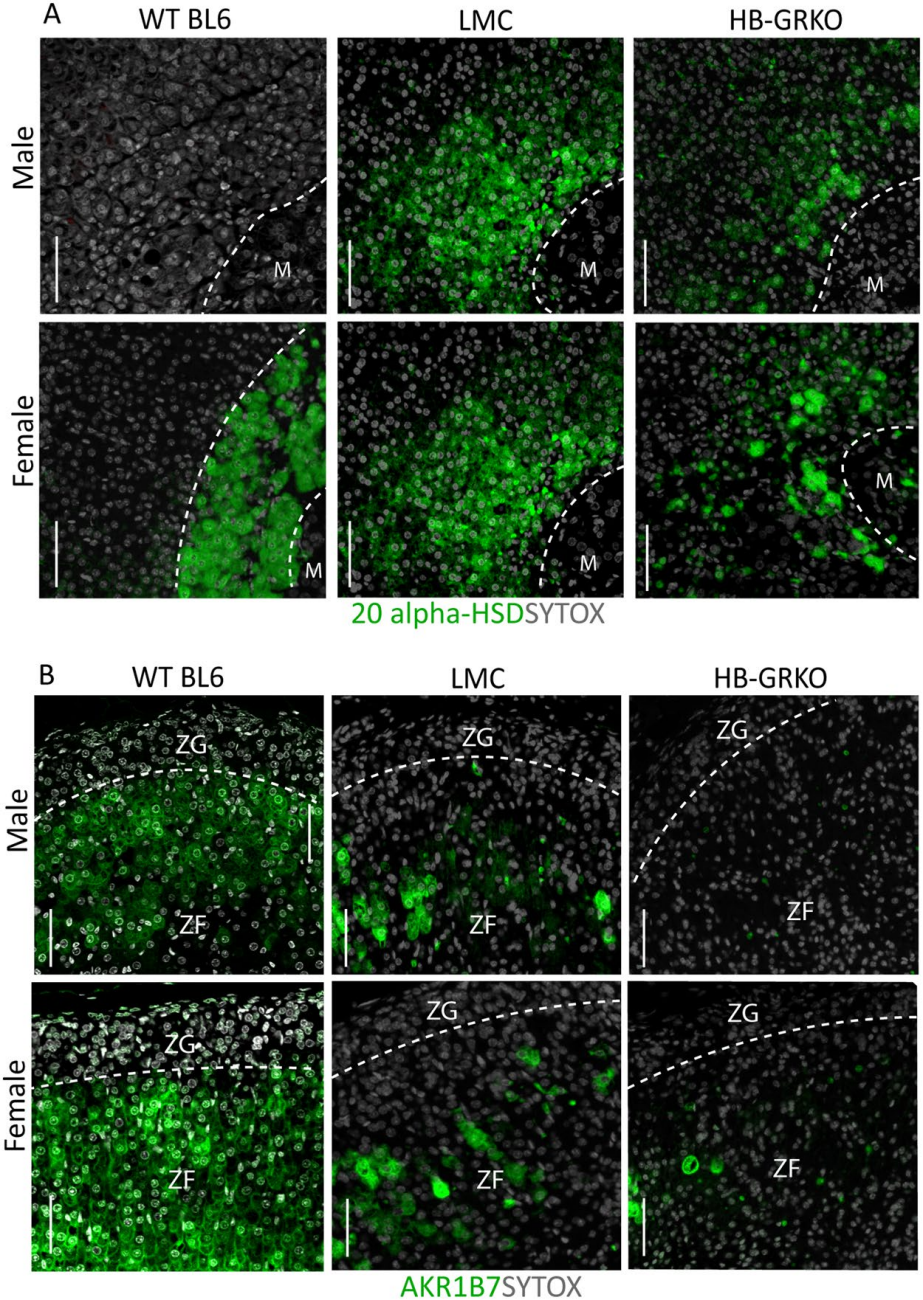
HB-GRKO LMC and HB-GRKO have disruption to adrenocortical markers. (**A**) Immunohistochemical localisation of 20 alpha-HSD revealed the presence of X-zone cells in both male HB-GRKO LMC and HB-GRKO mice compared to WT BL6 in which no expression is normally observed. Female HB-GRKO LMC and HB-GRKO mice show a disrupted 20 alpha-HSD localisation throughout the cortex, compared to BL6 controls that have a tightly packed X-zone at the cortex medulla boundary. (**B**) Immunohistochemical localisation of AKR1B7 revealed disruption in both male HB-GRKO LMC and HB-GRKO mice compared to WT BL6 mice, with less AKR1B7 positive cells being observed. Immunohistochemical localisation of AKR1B7 revealed disruption in both female HB-GRKO LMC and HB-GRKO mice compared to WT BL6 mice, with fewer AKR1B7 positive cells being observed. Abbreviations; ZG = zona glomerulosa, ZF = zona fasciculata. Scale bars 50 µm.

Analysis of X-zone marker 20-alpha-hydroxysteroid dehydrogenase (20 alpha-HSD)^19^ localization revealed ectopic 20 alpha-HSD positive cells in LMCs and HB-GRKO males, compared to WT BL6 males who normally do not express this marker in adulthood. 20 alpha-HSD localisation in females revealed dispersed positive cells throughout the cortex compared to the compact X-zone observed in WT BL6 females (Fig. 6A). Localization of aldo-keto reductase family 1, member B7 (AKR1B7), a well described zona fasciculata marker^20^, showed disruption in both male and female HB-GRKO and LMCs, compared to WT BL6 controls. LMCs displayed partial loss of AKR1B7 positive cells with HB-GRKO mice showing few positive cells (Fig. 6B).

**Figure 7:**
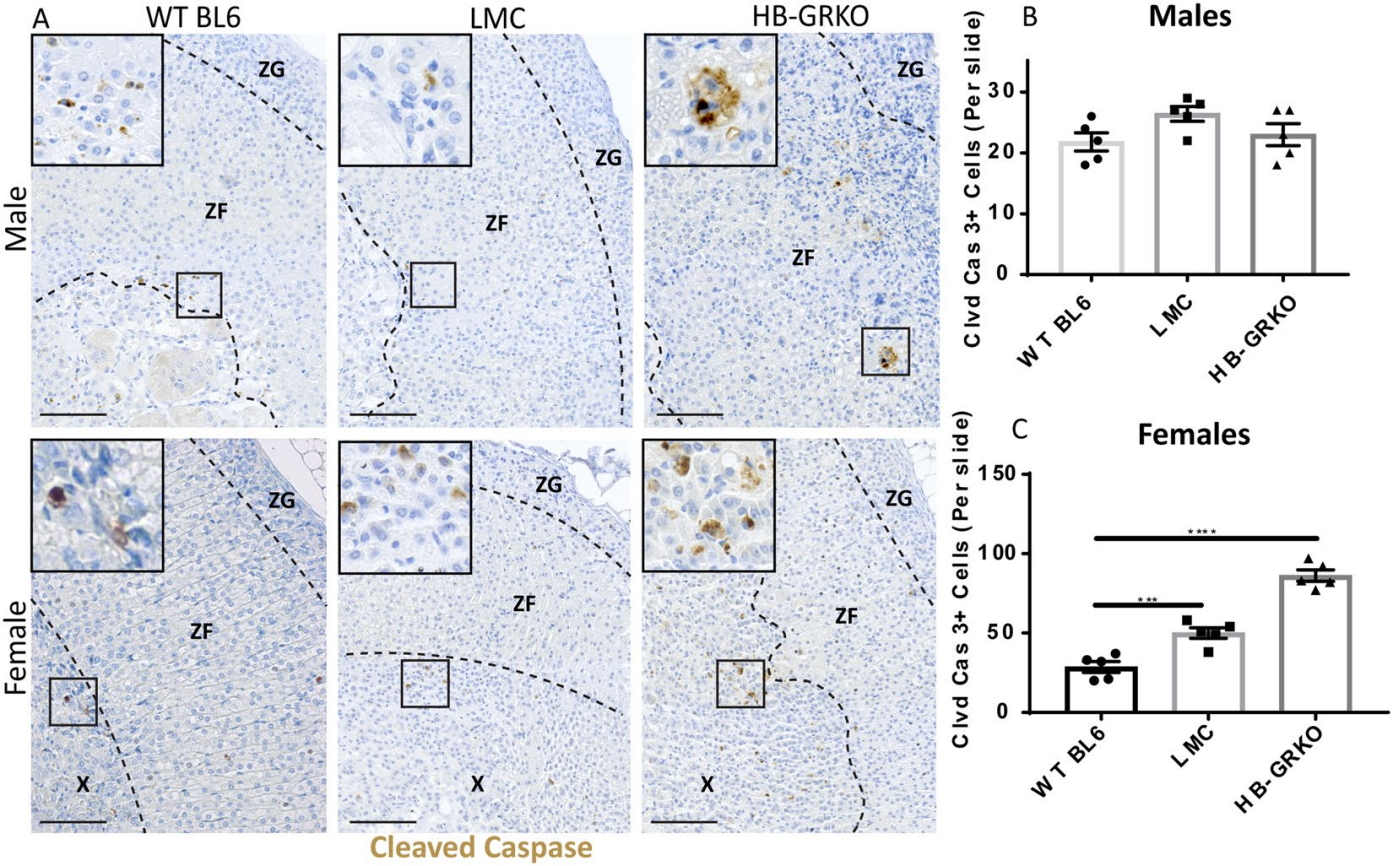
HB-GRKO mice show aberrant apoptosis throughout the adrenal cortex. Cell clearance from the cortex occurs at the cortex-medulla boundary which can be observed in WT Bl6 male and female controls via immunohistochemical analysis. HB-GRKO female Cleaved Caspase 3 protein localisation show abundant positive cells throughout the cortex. Inserts denoted by squares. (**B**) Cell counts show no changes between any of the male cohorts analysed. (**C**) Cleaved Caspase 3 counts reveals a significant increase in cell death throughout the cortex (One-way ANOVA; n = 5, ***p < 0.0001, ****p < 0.0001, Tukey’s post-hoc analysis, error bars SEM), consistent with the immunohistochemistry analysis. Scale bars 50 µm. Abbreviations; ZG = zona glomerulosa, ZF = zona fasciculata. Scale bars 100 µm.

To establish whether disruption to the adrenal cortex leads to changes in normal cell turnover, the apoptosis marker cleaved caspase 3 was examined. Normal apoptosis usually occurs at the cortex-medulla boundary; however, cleaved caspase 3 localization in HB-GRKO mice revealed many caspase-positive cells throughout the whole adrenal cortex in both males and females, indicating a large amount of aberrant cell death (Fig. 7). Together, these results show that loss of GR signalling in the hindbrain of both male and female mice, leads to disruption of structure of the adrenal glands. Similar disruption in the adrenal glands of LMCs (who retain hindbrain GR signalling) suggests that the adrenal phenotype is likely a secondary consequence of the stressed behaviour (excessive barbering of LMCs) exhibited by HB-GRKO cage-mates. This interpretation is consistent with prior studies linking a chronic stress response to degeneration of the adrenal gland^21^.

## Discussion

In this study we describe the initial phenotypic analysis of a novel mouse model for investigating the mechanisms contributing to stress and anxiety^11^. We confirm ablation of GR in the hindbrain with no off-target GR ablation in the adrenal cortex. We demonstrate that following GR ablation, stress behaviours presenting as excessive barbering and lack of cage exploration coupled with obsessive compulsive tendencies are observed. We show that disruption to hindbrain glucocorticoid signalling results in severe adrenal cortex disruption and an elevated stress response that is passed onto littermate controls. Excessive spinal curvature is also observed and could be attributed to the high levels of circulating corticosterone in males. These results highlight that GR signalling in the hindbrain is an important area of focus if we are to better understand the development of stress and anxiety in patients.

Characterising stress has proved difficult in humans due to the heterogeneity of the behaviours reported in patients with mood disorders^22^. Mouse models are challenging for these reasons, however, a range of indicators of stress behaviours have been well documented in rodents^6,14,23^. Our model demonstrates a number of characteristics linked to stress behaviour. Initial observations reveal severe hair removal via barbering. Although barbering is part of normal behaviour for rodents in bonding, dominance and cleaning^15^, if performed to excess, is indicative of elevated stress levels. The behaviours associated with barbering can also be further dissected to examine social encounters between the mice. These include dominant barbering, chasing and biting. By examining social interactions along with molecular mechanisms, this can further strengthen the translatability to patients. Therefore, investigating a model that not only has a genetic manipulation, but the ability to examine disruption to social interactions, is extremely useful^13,14,23,24^. Although not surprising, GR knockout mice in this study were also able to impact littermate controls, adding an additional layer of complexity to analysing results. This phenomenon has been demonstrated in experiments investigating social stressors on mice that were found to transfer a stress phenotype between littermates^13^. Separation of genotypes at an early stage would be feasible (as the phenotype does not present until adulthood) to prevent impacting littermate controls, however this could potentially select for a more severe phenotype, as it was noted that cages with a higher proportion of GR knockout mice are ranked amongst the highest on the barbering scale. The behavioural phenotype we observed in HB-GRKO mice overlap with behaviours of other brain specific knockout mouse models for disorders investigating anxiety. Targeted ablation of serotonin receptor 1A^25^ demonstrates reduced exploration, fewer rearing attempts and sex dependent variations. Further to this, GABA_A_ receptor ablation results in a similar phenotype when examining anxiety^26^. This would suggest that the utility of this model would be best used to investigate anxiety related behaviours, however this would require further validation. To understand the mechanisms behind these behavioural anomalies it will be important to examine the exact hindbrain structures that are not only expressing the Cre transgene but which of these hindbrain structures are expressing GR. It has been previously shown that GR mRNA is expressed in the hindbrain^6^ and immunohistochemistry studies have noted expression of GR in hindbrain structures including purkinje cells (GABAaergic neurones) and glial cells (nervous sytsem supporting cells)^27^. Furthermore, papers examining *Cyp11a1* activity in the brain have noted expression in both glial and purkinje cells^28,29^. These are potentially a focus for future research^30,31^, and teasing out the specific role of different cell-types in the hindbrain will likely require the use of cell-specific Cre lines.

The fluctuations we observe in body weight in male and female mice are also observed in patients with stress and anxiety disorders. It has also been shown that there are sex dependant differences in the response to stress^32^. The variation we observe in weight and corticosterone levels recapitulate this, highlighting that HB-GRKO mice are a translatable model to not only investigate stress but also the sex dependant differences in stress pathology. However, collection time of females could have potentially contributed to not detecting a difference in corticosterone. Corticosterone values can vary widely during oestrous and these females were not collected at a designated stage of the oestrous cycle^33^. Synchronising of oestrous cycles could potentially provide a more accurate result. Furthermore, sampling additional time points throughout the day could determine if cyclical corticosterone is perturbed. This would be particularly significant as changes in circadian rhythms have also been implicated in mood disorders^34^. Animals collected in this study were culled via CO_2_ inhalation and under minor stress conditions, however, controls were collated under the same conditions and therefore any changes are relative to controls. Further work could use trunk blood collection to determine more subtle changes in corticosterone between the groups.

Fluctuations in cortisol (in humans) or corticosterone (in rodents) have been shown to impact bone density and formation. The significant increase in spine curvature observed in both HB-GRKO animals is indicative of elevated corticosterone. However, this change in spinal curvature was seen in both males and females, with an increase in corticosterone only observed in males. Furthermore, there are no changes in spine curvature in male littermate controls despite elevated corticosterone levels. Glucocorticoids are well known for impacting bone density and formation^35^, however *Cyp11a1* has recently been shown to be present in bone tissue^36^. In this instance, there could be the potential that there is GR targeting in bone in HB-GRKO mice which could be attributing to the loss in bone structure, this would require further investigation.

Interrogation of adrenal morphology revealed severe disruption in male and female HB-GRKO mice with disorganisation of cortex zones, loss of structure in the zona fasciculata and pockets of vacuolisation and hyperplasia. As per the barbering phenotype, adrenal morphology was also impacted in littermate controls. Although this transfer of stress phenotype has been previously documented, we wanted to ensure that it was not a result of the Cre targeting or insertion of *loxP* sites. Analysis of GRflox mice showed no abnormal adrenal morphology. This demonstrates that the littermate controls are being affected by the HB-GRKO mice, suggesting a feedforward mechanism. Building on this, it would be important to investigate GR expression in the brain of littermate controls to see if GR is impacted and would also provide an interesting comparison between naturally induced stress and genetic manipulation induced stress.

Experiments examining chronic stress and its impact on the HPA axis has focused primarily on characterizing chronic stress-induced alterations in the brain. This central focus has occurred in part because the observed brain changes resemble those that are believed to arise in some types of stress-related psychiatric disorders, such as depression and anxiety. However, the brain works in fine balance with other endocrine organs such as the adrenal in mediating and dealing with stress. Although there is little research in mouse models as to the impact of chronic stress on the adrenals, this is more extensively researched in rat adrenals. A study conducted by Ulrich-Lai *et al.* highlighted that chronic stress induced by restraint in male rats, results in adrenal hyperplasia and hypertrophy to the adrenal cortex in a zone specific manner^21^. This is in line with what is observed in HB-GRKO mice with enlarged adrenals and hyperplasia observed throughout the cortex. This study also noted that there was a decrease in zona glomerulosa size due to hypoxia. Measurements of cortex zones in HB-GRKO mice would also be useful to determine if there is an impact to specific zone size as a result of chronic stress as this could have further ramifications for steroid production from the adrenal gland.

In HB-GRKO mice, X-zone cells were maintained in males and females in adulthood and not constrained to a defined zone at the medulla boundary. Instead, these cells are widely distributed throughout the adrenal cortex. This migration of X-zone cells throughout the cortex has been associated with pathologies such as primary pigmented nodular adrenocortical disease and Cushing’s syndrome^37^. Furthermore, the distribution of these cells through the cortex has implications for cell turnover and definitive cortex regulation. AKR1B7, known for its role in detoxifying products from cholesterol cleavage^20^, reveals a loss in protein localisation which could result in toxic products building up in the adrenal cortex and ultimately lead to further damage. Unsurprisingly, analysis of cleaved caspase localisation revealed many apoptotic cells throughout the entire adrenal cortex in HB-GRKO mice, highlighting considerable cell death and damage. This could be attributed to the over stimulation of the adrenal cortex by the hindbrain resulting in cell death and loss of AKR1B7. This data combined shows distinct disruption to adrenal morphology and function as a result of GR disruption in the hindbrain further proving its utility as a model of stress.

The data herein describes that ablation of GR in the hindbrain results in increased barbering, stressed cage behaviour and impacted corticosterone levels. Additionally, it builds on previous literature that describes a relationship between stress and the dysregulation of the HPA axis leading to adrenal cortex damage. These results highlight a new model that could provide new insights into the development of stress and anxiety.

## Material and Methods

All mice used in experiments were under a strict standard of care and experimental planning covered by licensed approval from the UK Home Office and adhere to the ARRIVE (Animal Research: Reporting of *In Vivo* Experiments) guidelines^38^, (License number 80/7704).

### Targeted ablation of GR from the hindbrain using Cyp11a1-GC Cre

To specifically ablate GR from the hindbrain, Cre/*loxP* technology was used. Female C57BL/6 mice carrying a random insertion of the *Cyp11a1*-GC Cre^11^ were mated to C57BL/6 male mice homozygous (Hom) for floxed GR^11^. The first generation resulted in offspring heterozygous (Het) for GRflox that were either Cre+ or Cre−. For total hindbrain GR ablation Cre+ GR heterozygous males were again bred to C57BL/6 female mice homozygous for floxed GR resulting in the following offspring: Cre− Hom termed ‘littermate control’ (LMC), Cre− Het, termed ‘Het littermate control’ (Het LMC), Cre+ Hom, termed ‘Hindbrain GR knockout’ (HB-GRKO), Cre+ Het, termed ‘Het hindbrain GR knockout’ (Het HB-GRKO). In addition to these mice, an external control group of wild type (WT) male and female mice that have had no interaction with GR knockout mice but had been housed on the same shelf and under the same conditions. These animals were termed ‘WT Bl6’.

### PCR genotyping of mice

Mice were genotyped for the inheritance of Cre recombinase as previously described^40^. PCR amplification products were resolved using QIAxcel capillary system (QIAGEN, Crawley, United Kingdom). An amplicon of 102 bp indicated the inheritance of the Cre recombinase transgene. Mice were also genotyped for the inheritance of floxed GR using primer sequences forward GGCATGCACATTACTGGCCTTCT, reverse 1 GTGTAGCAG CCAGCTTACAGGA and reverse 2 CCTTCTCATTCCATGTCAGCATGT. Expected band sizes were 2.5 kb for wild type GR and 500 bp for recombined GR.

### Determination of genomic ablation of GR

DNA was isolated from a piece of frozen hindbrain tissue using a genomic DNA kit (Gen-probe Life Sciences Ltd, UK) according to the manufacturer’s instructions and subjected to PCR amplification using primers GCAGCACATAGGGCATCTTC and AGCCAGCTTACAGGATAGCC. PCR amplification products were resolved using QiaXcel capillary system (Qiagen UK). An amplicon of 2.5 kb represented floxed GR and an amplicon of 500 bp represented recombination between *loxP* sites of GR in exon 3.

### Tissue collection and processing

Mice were culled by inhalation of carbon dioxide and subsequent cervical dislocation at between d90 and d100. Body weight was measured and adrenals were removed and weighed. Tissues were fixed in Bouin’s fixative (Clin-Tech, Guildford, UK) for 4 hours (adrenals). Bouin’s-fixed tissues were processed and embedded in paraffin wax, and 5 µm sections were used for histological analysis. Sections of adrenals were stained with haematoxylin and eosin using standard protocols and examined for histological abnormalities.

### Immunohistochemistry

Immunolocalization was performed either by a single antibody colourimetric (DAB) immunostaining method, as described previously^41^, a single or double antibody tyramide fluorescent immunostaining method, as described previously^11,42^, or using an automated Bond immunostaining method, as described previously^41^, Exceptions to this protocol are the H_2_O_2_ concentration washes performed at 3% H_2_0_2_ TBS. Antibodies used are listed in Table 1. A minimum of five individual sections for each genotype were immunostained in each experiment.

**Table 1:** Immunohistochemistry performed in this study, listing antibody source and method used.

Protein stained for	Method	Primary antibodies
PCNA	DAB	Sigma #P-8825
AKR1B7	Single fluorescence	Santa Cruz Biotechnology #sc-27763
HSD20alpha	Single fluorescence	Aviva Systems Biology #40002
GR	DAB	Cell Signalling #12041
GR/GFP	Double fluorescence	Cell Signalling #12041, Thermofisher scientific #a11122
CASP3	Bond	CASP3: Abcam #ab4051

###  PET/CT Scan analysis of spinal curvature

Interrogation of spinal curvature was achieved via CT scanning. Whole body micro CT images of mice was acquired. 8 groups of animals were be included in this study with 5 animals per group between male and female controls and knockout mice. Collected CT images were reconstructed and analysed using PMOD software. Mice were carefully positioned head first prone in the scanner bed for collection of coronal and sagittal plane radiographs using a nanoPET/CT scanner (Mediso, Hungary) and the following settings: side or top view, X-ray energy of 50 kVp, exposure time of 300 ms and maximum field of view. Coronal and sagittal plane radiographs were used for animal positioning for high-resolution microCT imaging, which was acquired using the following parameters: semi-circular full trajectory, 720 projections, maximum zoom, tube voltage of 50 kVp, exposure time of 300 ms, binning of 1:4. Nucline software (Mediso, Hungary) was used to reconstruct microCT images using the following parameters: voxel size medium, slice thickness medium and cosine filter with 100% cut-off (combined voxel resolution: isotropic 251 μm). The Nucline software was also used to assess gross anatomical measurements and to measure the magnitude of the largest scoliotic and thoraco-lumbar kyphotic curves, according to the Cobb method^43^.

### Barbering Scale

To determine if barbering observed falls out-with normal cage behaviour, hair loss was graded as follows; No hair loss = 0, removal of whiskers = 1, removal of whiskers and facial hair = 2, removal of whiskers, facial hair that extends to head = 3, removal of whiskers and hair on the face, head and back = 4 and removal of whiskers and hair on the face, head, back and stomach = 5. Hair loss noted from 0–2 was deemed to fall within the normal range of barbering that would be expected in WT animals, any barbering scored 3 or higher was deemed abnormal and more severe than would be expected under normal conditions^14,15^.

### Extraction of steroid hormones from plasma

Immediately after culling (after CO2 and before cervical dislocation), blood was collected from C57BL6, LMC and HB-GRKO mice via cardiac puncture with a syringe and needle with a wide bevel to reduce lysis, blood was collected in EDTA coated tubes to prevent coagulation. Animals were allowed to settle in the new room to allow normalization of corticosterone levels before collection. Plasma was separated by centrifugation and stored at −80 °C for future analysis.

Analysis of androgens and corticosteroids in mouse serum was achieved by isotope-dilution TurboFlow-LC-MS/MS. The method was developed for quantification of Δ4-androstenedione (Adione), testosterone (T), 17α-hydroxyprogesterone (17-OHP), progesterone, corticosterone and estrone 3-sulfate (E1-S) in human serum^44^ and was used without modifications for mouse serum in the present study. For validation, and in addition to human serum control material made of human serum spiked in different levels, control materials of two mouse serum pools were used (Supplementary Table 1A). These samples were expected to have low and high testosterone levels which permitted the preparation of four different mouse control samples; two times 300 μ1 of each of the mouse serum pools were spiked in low and high levels by adding respectively 15 μL and 35 μL of calibration standard 6, before further sample preparation. All analysis of control material, linearity and matrix effects in mouse serum and all mouse serum samples were analysed in three batches. Each of these batches included standards for calibration curves, randomized samples, two blanks, human control material (two times three different concentrations) along with one of each of the four mouse control samples. The inter-day variation for mouse serum control material expressed as the relative standard deviation (RSD) was ≤11% and the recovery was >90% for all steroids in both spike levels (Supplementary Table 1B).

### Statistical analysis

Statistical analysis was performed using GraphPad Prism (version 7; GraphPad Software Inc., San Diego, CA, USA) using a two-tailed unpaired t test (if comparing two groups), a one-way ANOVA with Tukeys post-hoc test (if comparing multiple groups groups). Values are expressed as means ± S.E.M.

## Supplementary information

Supplementary Figure 1, 2, 3, 4

## Data Availability

All data generated as part of this study has been included in this manuscript in either the main body or in Supplementary Figures.

## References

[1] McEwen BS (2005). Glucocorticoids, depression, and mood disorders: structural remodeling in the brain. Metabolism..

[2] Young EA, Haskett RF, Murphy-Weinberg V, Watson SJ, Akil H (1991). Loss of glucocorticoid fast feedback in depression. Archives of general psychiatry..

[3] Holsboer F (2000). The corticosteroid receptor hypothesis of depression. Neuropsychopharmacology..

[4] Anacker C, Zunszain PA, Carvalho LA, Pariante CM (2011). The glucocorticoid receptor: pivot of depression and of antidepressant treatment?. Psychoneuroendocrinology..

[5] Ehlert U, Gaab J, Heinrichs M (2001). Psychoneuroendocrinological contributions to the etiology of depression, posttraumatic stress disorder, and stress-related bodily disorders: the role of the hypothalamus–pituitary–adrenal axis. Biological Psychology..

[6] Zhang R (2010). Role of glucocorticoids in tuning hindbrain stress integration. Journal of Neuroscience..

[7] Ulrich-Lai YM, Herman JP (2009). Neural regulation of endocrine and autonomic stress responses. Nature Reviews Neuroscience..

[8] Dedic, N. Genetic dissection of CRH-controlled neurocircuitries of stress. PhD diss., https://mediatum.ub.tum.de/1225581 (Technische Universität München, 2015)

[9] Chen L-S, Eaton WW, Gallo JJ, Nestadt G (2000). Understanding the heterogeneity of depression through the triad of symptoms, course and risk factors: a longitudinal, population-based study. Journal of Affective Disorders..

[10] Schmidt M (2006). Differential disinhibition of the neonatal hypothalamic–pituitary–adrenal axis in brain‐specific CRH receptor 1‐knockout mice. European Journal of Neuroscience..

[11] O’Hara L, York JP, Zhang P, Smith LB (2014). Targeting of GFP-Cre to the mouse Cyp11a1 locus both drives cre recombinase expression in steroidogenic cells and permits generation of Cyp11a1 knock out mice. PloS one..

[12] Urani A, Chourbaji S, Gass P (2005). Mutant mouse models of depression: candidate genes and current mouse lines. Neuroscience & Biobehavioral Reviews..

[13] Beery AK, Kaufer D (2015). Stress, social behavior, and resilience: insights from rodents. Neurobiology of stress..

[14] Kalueff A, Minasyan A, Keisala T, Shah Z, Tuohimaa P (2006). Hair barbering in mice: implications for neurobehavioural research. Behavioural Processes..

[15] Garner JP, Dufour B, Gregg LE, Weisker SM, Mench JA (2004). Social and husbandry factors affecting the prevalence and severity of barbering (‘whisker trimming’) by laboratory mice. Applied Animal Behaviour Science..

[16] Rodgers R, Cole J (1993). Influence of social isolation, gender, strain, and prior novelty on plus-maze behaviour in mice. Physiology & behavior..

[17] Lee SW (2007). Analysis of accuracy of kyphotic angle measurement for vertebral osteoporotic compression fractures. Journal of Clinical Neuroscience..

[18] Ross E, Marshall-Jones P, Friedman M (1966). Cushing’s syndrome: diagnostic criteria. QJM..

[19] Hershkovitz L, Beuschlein F, Klammer S, Krup M, Weinstein Y (2007). Adrenal 20α-hydroxysteroid dehydrogenase in the mouse catabolizes progesterone and 11-deoxycorticosterone and is restricted to the X-zone. Endocrinology..

[20] Martinez A (2001). Physiological functions and hormonal regulation of mouse vas deferens protein (AKR1B7) in steroidogenic tissues. Chemico-Biological Interactions..

[21] Ulrich-Lai YM (2006). Chronic stress induces adrenal hyperplasia and hypertrophy in a subregion-specific manner. American Journal of Physiology-Endocrinology and Metabolism..

[22] Dedic, N., Walser, S. M. & Deussing, J. M. Mouse models of depression. *Psychiatric Disorders—Trends and Developments* (Uehara, Ed.) 185–222 (Croatia 2011).

[23] Denmark A (2010). The effects of chronic social defeat stress on mouse self-grooming behavior and its patterning. Behavioural Brain Research..

[24] Bailey, K. R. & Crawley, J. N. Anxiety-related behaviors in mice (ed. Buccafusco J. J.), CRC Press (USA 2009). 21204329

[25] Ramboz S (1998). Serotonin receptor 1A knockout: an animal model of anxiety-related disorder. Proceedings of the National Academy of Sciences..

[26] Crestani F (1999). Decreased GABA A-receptor clustering results in enhanced anxiety and a bias for threat cues. Nature Neuroscience..

[27] Furukawa A, Miyatake A, Ohnishi T, Ichikawa Y (1998). Steroidogenic acute regulatory protein (StAR) transcripts constitutively expressed in the adult rat central nervous system: colocalization of StAR, cytochrome P‐450SCC (CYP XIA1), and 3β‐hydroxysteroid dehydrogenase in the rat brain. Journal of Neurochemistry..

[28] Kushida A, Tamura H (2009). Retinoic acids induce neurosteroid biosynthesis in human glial GI-1 Cells via the induction of steroidogenic genes. Journal of Biochemistry..

[29] Tsutsui K (2008). Neurosteroids in the Purkinje cell: biosynthesis, mode of action and functional significance. Molecular Neurobiology..

[30] Pelletier G (2010). Steroidogenic enzymes in the brain: morphological aspects. Progress in Brain Research..

[31] Tsutsui K, Ukena K, Takase M, Kohchi C, Lea RW (1999). Neurosteroid biosynthesis in vertebrate brains. Comparative Biochemistry and Physiology Part C: Pharmacology, Toxicology and Endocrinology..

[32] Verma R, Balhara YPS, Gupta CS (2011). Gender differences in stress response: Role of developmental and biological determinants. Industrial Psychiatry Journal..

[33] Atkinson HC, Waddell BJ (1997). Circadian variation in basal plasma corticosterone and adrenocorticotropin in the rat: sexual dimorphism and changes across the estrous cycle. Endocrinology..

[34] Chung S, Son GH, Kim K (2011). Circadian rhythm of adrenal glucocorticoid: its regulation and clinical implications. Biochimica et Biophysica Acta (BBA)-Molecular Basis of Disease..

[35] Tauchmanova L (2006). Bone demineralization and vertebral fractures in endogenous cortisol excess: role of disease etiology and gonadal status. The Journal of Clinical Endocrinology & Metabolism..

[36] Rodriguez-Sanz M (2015). CYP11A1 expression in bone is associated with aromatase inhibitor-related bone loss. Journal of Molecular Endocrinology..

[37] Sahut-Barnola I (2010). Cushing’s Syndrome and fetal features resurgence in adrenal cortex–specific prkar1a knockout mice. PLoS Genetics..

[38] Kilkenny C, Browne W, Cuthill IC, Emerson M, Altman DG (2010). Animal research: reporting *in vivo* experiments: the ARRIVE guidelines. British Journal of Pharmacology..

[39] De Gendt K (2004). A Sertoli cell-selective knockout of the androgen receptor causes spermatogenic arrest in meiosis. Proceedings of the National Academy of Sciences..

[40] Welsh M, Saunders PT, Atanassova N, Sharpe RM, Smith LB (2009). Androgen action via testicular peritubular myoid cells is essential for male fertility. The FASEB Journal..

[41] O’Hara L, Welsh M, Saunders PT, Smith LB (2010). Androgen receptor expression in the caput epididymal epithelium is essential for development of the initial segment and epididymal spermatozoa transit. Endocrinology..

[42] O’Hara L, Smith LB (2012). Androgen receptor signalling in Vascular Endothelial cells is dispensable for spermatogenesis and male fertility. BMC Research Notes..

[43] COBB JR (1948). Outline for the study of scoliosis. Instructional Course Lectures..

[44] Søeborg T, Frederiksen H, Johannsen TH, Andersson A-M, Juul A (2017). Isotope-dilution TurboFlow-LC-MS/MS method for simultaneous quantification of ten steroid metabolites in serum. Clinical Chimical Acta..

