# Supplementary material for: Ablation of glucocorticoid receptor in the hindbrain of the mouse provides a novel model to investigate stress disorders
Source: Sci Rep. 2019 Mar 1;9:3250. doi: 10.1038/s41598-019-39867-y (PMC6397323; doi:10.1038/s41598-019-39867-y)
Supplement: Supplementary file 1 — Supplementary Figure 1, 2, 3, 4 [file 41598_2019_39867_MOESM1_ESM.pdf]

**SREP-18-32522A**

**Ablation of glucocorticoid receptor in the hindbrain of the mouse provides a novel model to investigate stress disorders**

Anne-Louise Gannon[1, 5], Laura O'Hara[1, 2], J. Ian Mason[1], Diane Rebourcet, [1, 5], Sarah Smith[1], Adriana Traveres[3], Carlos Jose Alcaide-Corral[3], Hanne Frederiksen[4] Anne Jørgensen[4], Laura Milne[1], Rod T. Mitchell[1] and Lee B. Smith[1, 5*]

[1] MRC Centre for Reproductive Health, University of Edinburgh, The Queen's Medical Research Institute, 47 Little France Crescent, Edinburgh, EH16 4TJ. UK. [2] Centre for Discovery Brain Sciences, Hugh Robson Building, George Square, Edinburgh, EH8 9XD, UK.[3] Edinburgh Preclinical Imaging, College of Medicine and Veterinary Medicine, University of Edinburgh, EH16 4TJ. UK. [4] Department of Growth and Reproduction, Rigshospitalet, University of Copenhagen, Denmark, International Centre for Research and Research Training in Endocrine Disruption of Male Reproduction and Child Health (EDMaRC), Rigshospitalet, Denmark. [5] School of Environmental and Life Sciences, Faculty of Science, University of Newcastle, Callaghan, 2308, NSW, Australia

* To whom correspondence should be addressed: Professor Lee Smith, MRC Centre for Reproductive Health, University of Edinburgh, The Queen's Medical Research Institute, 47 Little France Crescent, Edinburgh EH16 4TJ, UK. Tel: +44 (0)131 242-9111 Email: Lee.Smith@ed.ac.uk

This work was funded by a Medical Research Council Program Grant Award (MR/N002970/1) (to LBS)

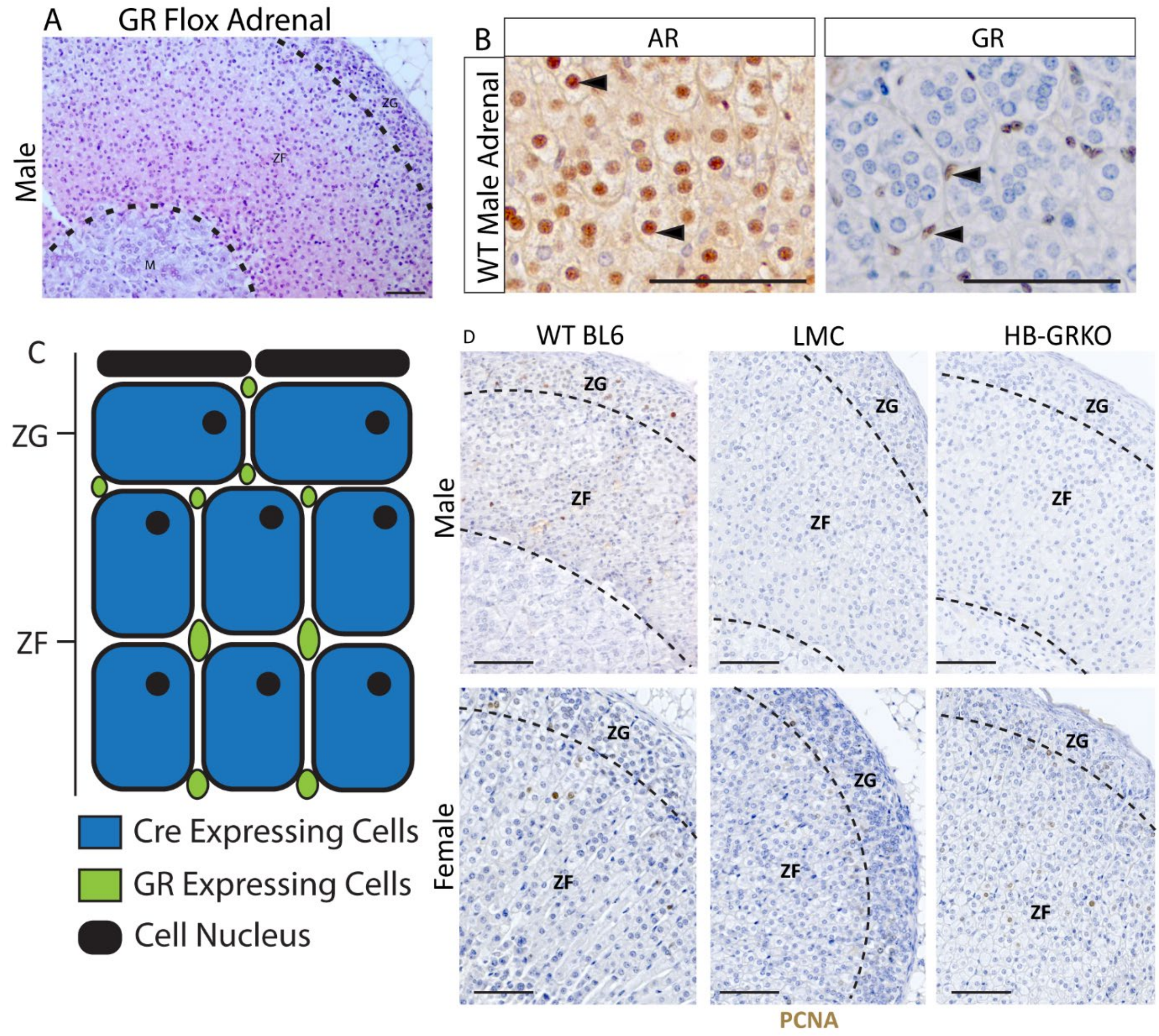


**Supplementary Figure 1.**

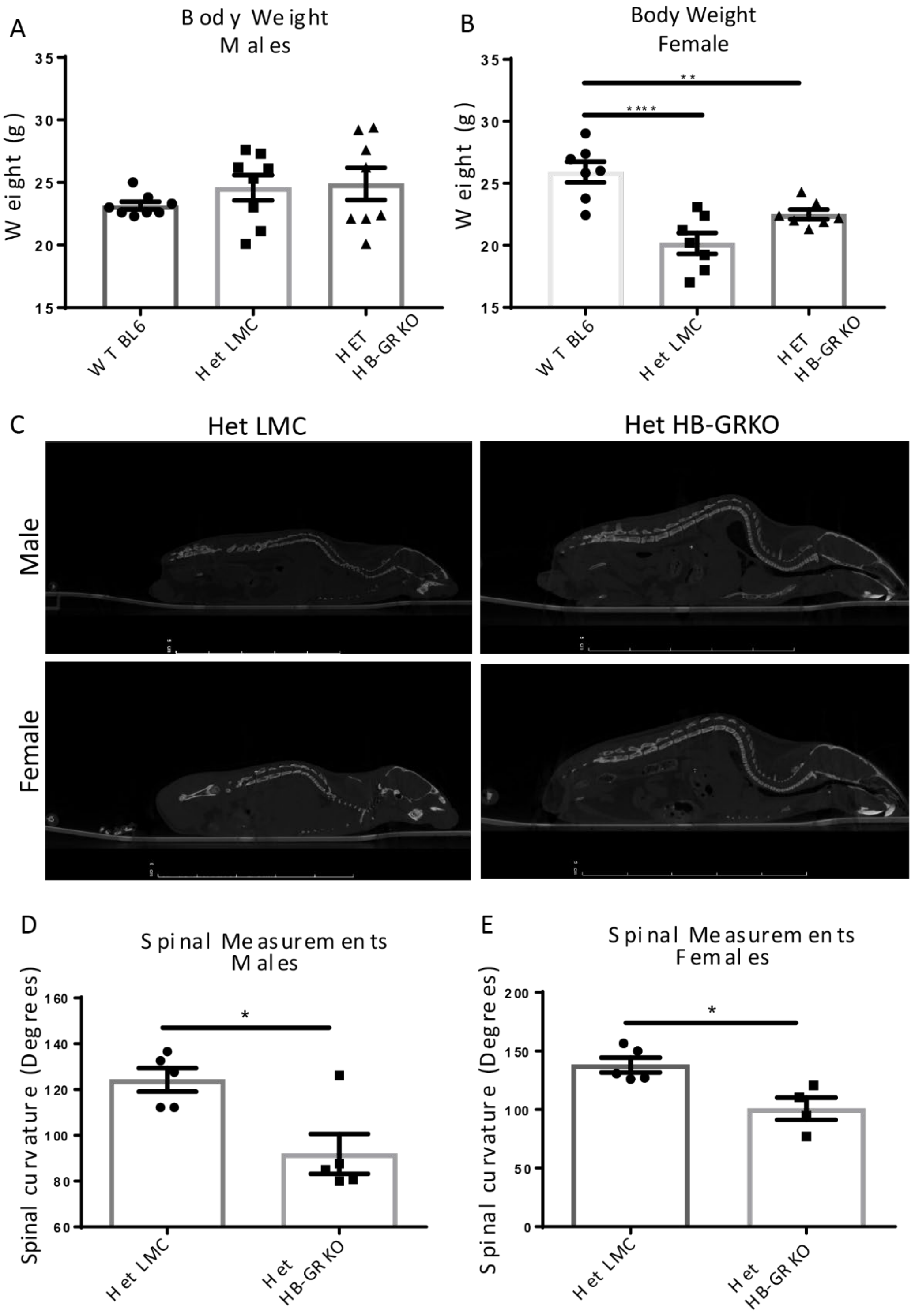


**Supplementary Figure 2.**

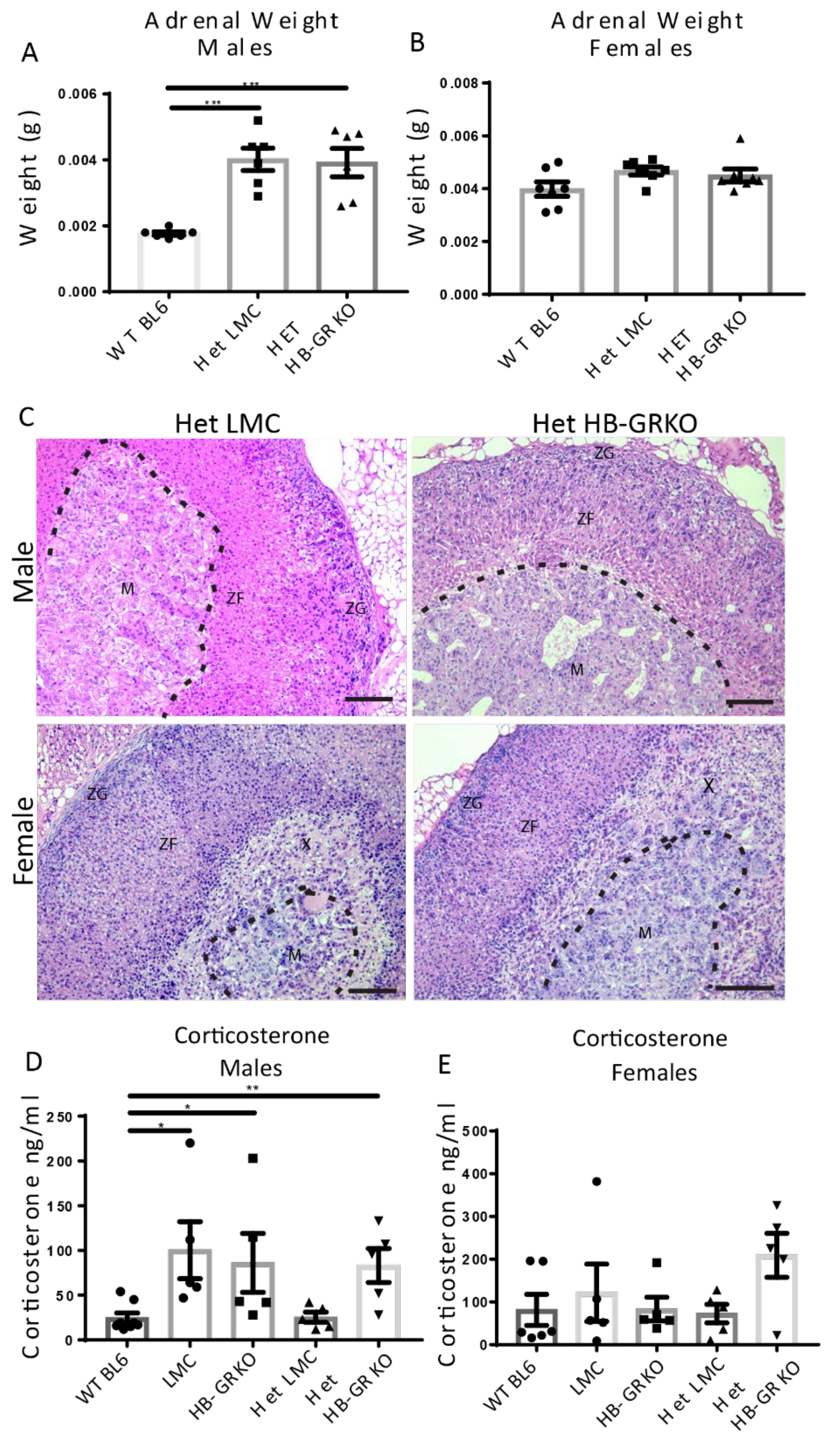


**Supplementary Figure 3.**

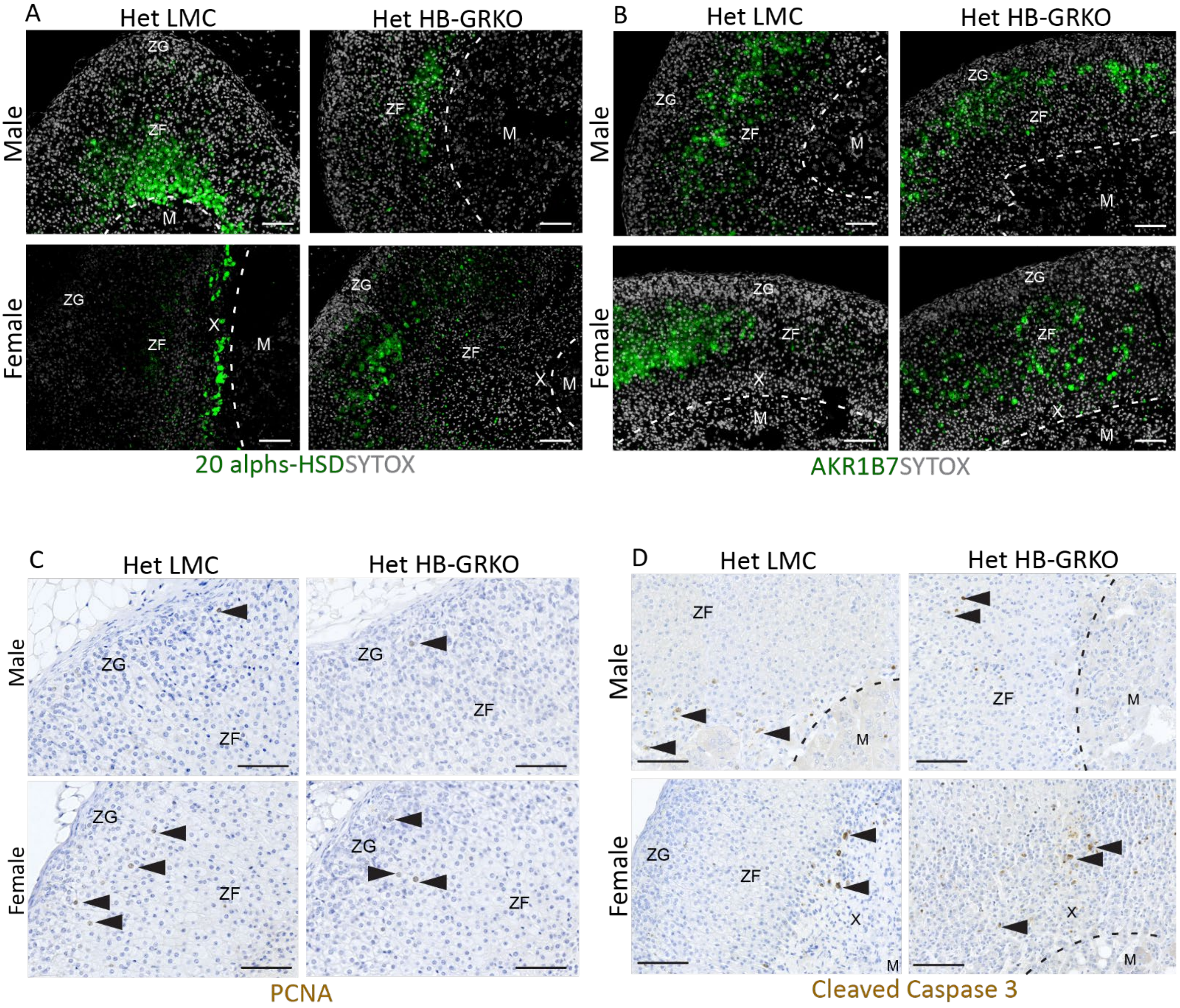


**Supplementary Figure 4.**

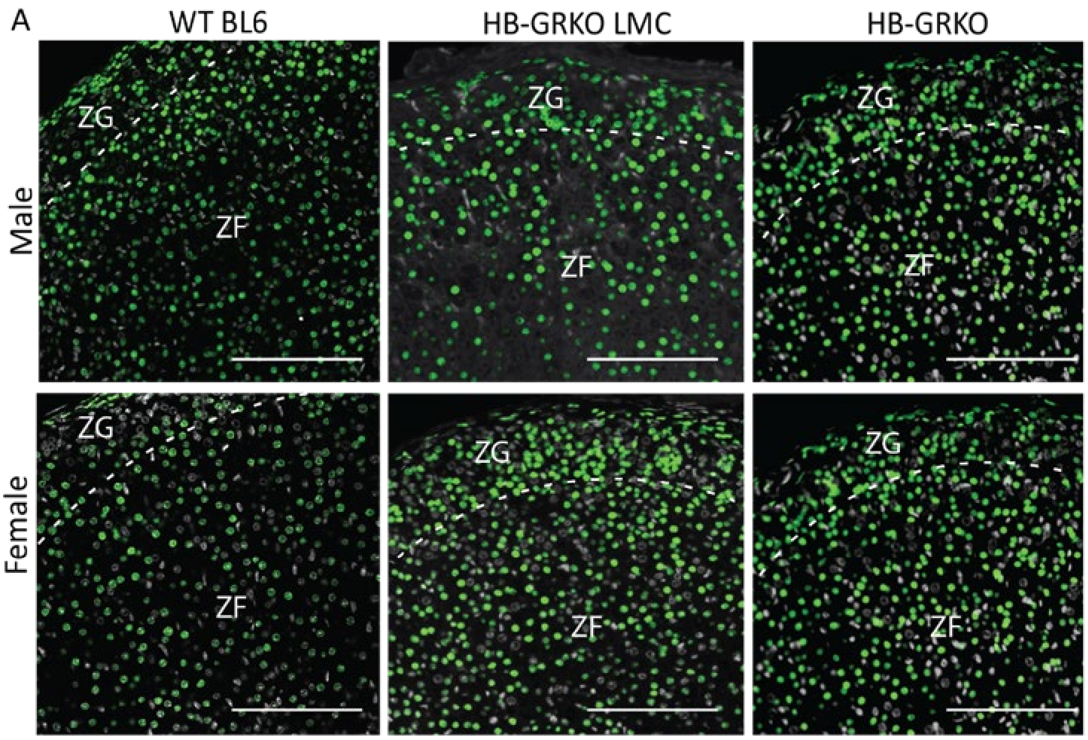


**Supplementary Figure 5.**

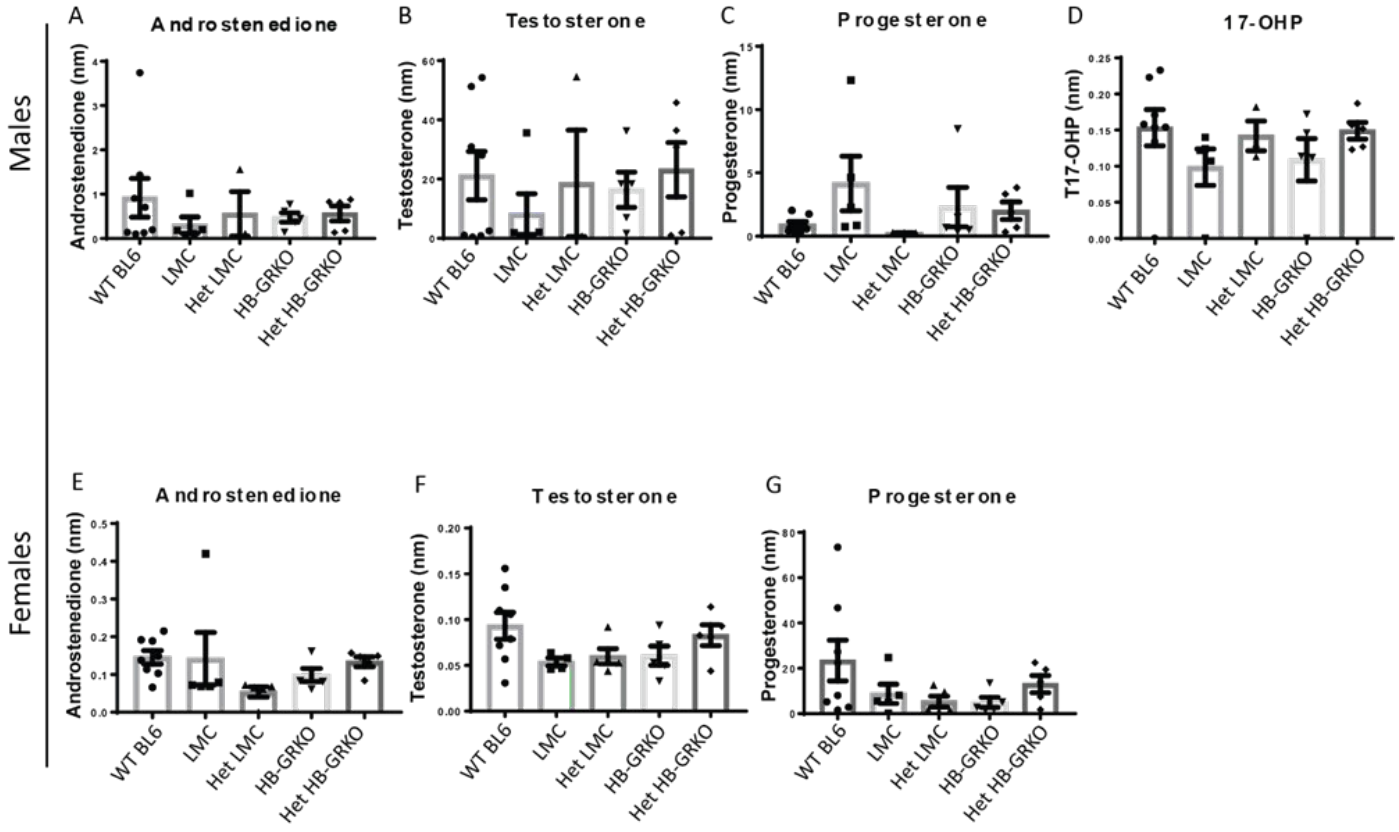


**Supplementary Figure 6.**

A

| | LOQ (nM) | Range (nM) |
|---|---|---|
| Estrone 3-sulfate | 0.026 | LOQ-10 |
| Cortisone | 0.19 | LOQ-112 |
| Cortisol | 1.9 | LOQ-794 |
| dehydroepiandrosterone sulfate (DHEAS) | 19 | LOQ-3000 |
| Corticosterone | 0.1 | LOQ-144 |
| 11-deoxycortisol | 0.017 | LOQ-40 |
| Δ4-androstenedione | 0.042 | LOQ-1746 |
| Testosterone | 0.012 | LOQ-1732 |
| 17α-hydroxyprogesterone | 0.1 | LOQ-1513 |
| Progesterone | 0.036 | LOQ-500 |

B

| | Serum #72 Q Low | | | Serum #72 Q High | | | Serum #73 Q Low | | | Serum #73 Q High | | |
|---|---|---|---|---|---|---|---|---|---|---|---|---|
| | mean (nM) | RSD (%) | recovery (%) | mean (nM) | RSD (%) | recovery (%) | mean (nM) | RSD (%) | recovery (%) | mean (nM) | RSD (%) | recovery (%) |
| Estrone 3-sulfate | 0.43 | 7.0 | 105 | 0.94 | 5.7 | 99 | 0.39 | 8.7 | 96 | 0.92 | 8.9 | 96 |
| Cortisone | 4.63 | 0.87 | 100 | 10.6 | 7.6 | 98 | 4.20 | 4.1 | 91 | 10.4 | 3.4 | 97 |
| Cortisol | 35.3 | 8.1 | 102 | 81.0 | 2.3 | 100 | 32.3 | 0.90 | 93 | 77.7 | 1.7 | 96 |
| dehydroepiandrosterone sulfate | 720 | 7.5 | 104 | 1652 | 2.7 | 102 | 671 | 1.1 | 97 | 1584 | 1.4 | 98 |
| Corticosterone | 38.0 | 7.3 | 107 | 46.5 | 12 | 109 | 18.3 | 8.7 | 103 | 25.5 | 0.54 | 98 |
| 11-deoxycortisol | 1.86 | 2.3 | 104 | 4.07 | 6.8 | 98 | 1.78 | 1.9 | 96 | 4.02 | 3.4 | 95 |
| Δ4-androstenedione | 2.13 | 0.74 | 102 | 4.72 | 4.2 | 97 | 3.39 | 6.8 | 94 | 5.98 | 3.0 | 93 |
| Testosterone | 5.62 | 1.1 | 111 | 7.71 | 3.1 | 99 | 49.3 | 3.0 | 106 | 51.4 | 3.0 | 98 |
| 17α-hydroxyprogesterone | 2.09 | 11 | 96 | 4.53 | 7.0 | 95 | 2.18 | 6.3 | 90 | 4.67 | 0.36 | 93 |
| Progesterone | 3.48 | 1.3 | 100 | 7.99 | 4.9 | 98 | 3.89 | 5.5 | 92 | 8.33 | 5.2 | 94 |

**Supplementary Table 1.**

## Supplementary Figure Legends

**Supplementary Figure 1.** (A) Morphology analysis of GR flox adrenals show no disruption to the adrenal cortex. N=4. (B) Immunolocalisation demonstrating AR localisation in large steroidogenic cells and GR localisation in between these cell populations. (C) Diagram depicting GR cells interspersed between Cre expressing cells. (D) Immunohistochemistry analysis of PCNA revealed no changes in proliferation in experimental cohorts compared to WT BL6. N=6. Scale bars 100µm. Abbreviations; ZG= zona glomerulosa, ZF= zona fasciculata, M=medulla.

**Supplementary Figure 2.** (A) Male body weights of heterozygous mice compared to an external WT BL6 cohort reveals no difference between the cohorts. There is no difference in weights in HB-GRKO LMC compared to BL6 controls. (B) Female body weights of heterozygous mice compared to an external WT BL6 cohort show a significant decrease in both in HB-GRKO LMC and HB-GRKO mice (one-way ANOVA; n=8, ****p<0.0001, **p<0.001 Tukey's post-hoc analysis, error bars SEM). (C) CT scans show that the spine in Het HB-GRKO mice have an increase in spinal curvature and show spinal collapse in HB-GRKO mice compared to littermate controls, N=5. (D, E) Measurement of spine angle confirms increase in curvature (one-way ANOVA; n=5, *p<0.05, *p<0.05, Tukey's post-hoc analysis, error bars SEM). Scale bar 5cm.

**Supplementary Figure 3.** (A) Analysis of male adrenal weight revealed an increase in weight in all cohorts compared to WT BL6 controls (One-way ANOVA; n=6-8, ***p<0.0001, ***p<0.0001, Tukey's post-hoc analysis, error bars SEM). (B) Analysis of female adrenal weight revealed no differences between any of the cohorts compared to WT BL6 controls. (C) Morphology analysis of male and female Het LMC and Het HB-GRKO adrenals display major disruption to the entire cortex. (D) Circulating corticosterone levels are elevated in both male HB-GRKO LMC and HB-GRKO mice compared to WT BL6 mice (One-way ANOVA; n=5-8, *p<0.05, *p<0.05, Tukey's post-hoc analysis, error bars SEM). This increase is also observed in Het HB-GRKO mice (One-way ANOVA; n=5-8, **p<0.001, Tukey's post-hoc analysis, error bars SEM), but not Het LMCs (E) Circulating corticosterone levels do not change in any female cohort compared to WT BL6 controls. N=5. Abbreviations; ZG= zona glomerulosa, ZF= zona fasciculata, X=X-zone, M=medulla. Scale bars 100µm.

**Supplementary Figure 4.** (A) Immunohistochemical localisation of 20 alpha-HSD reveals the presence of foetal X-zone cells in both male Het LMC and Het HB-GRKO mice. Although normally retained in the adult female cortex, Het HB-GRKO females show X-zone cells throughout the cortex and no longer confined to the cortex medulla boundary. (B) Immunohistochemical localisation of AKR1B7 revealed disruption in both male Het LMC and Het HB-GRKO mice, with fewer AKR1B7 positive cells being observed, this is recapitulated in female Het LMC and Het HB-GRKO mice. (C) Immunohistochemistry analysis of PCNA revealed no changes in proliferation in any male or female experimental cohorts compared to WT BL6 controls. (D) Cell clearance from the cortex occurs at the cortex-medulla boundary which can be observed in WT Bl6 controls via immunohistochemical analysis. HB-GRKO het male and female cleaved caspase 3 protein localisation show positive cells throughout the cortex. Abbreviations; ZG= zona glomerulosa, ZF= zona fasciculata, X=X-zone, M=medulla. N=5. Scale bars 100µm.

**Supplementary Figure 5.** (A) Immunohistochemical localisation of androgen receptor reveals no changes in localisation in any cohort analysed. Androgen receptor; Green, SYTOX; Grey. Abbreviations; ZG= zona glomerulosa, ZF= zona fasciculata, X=X-zone, M=medulla. N=5. Scale bars 100µm.

**Supplementary Figure 6.** (A-D) No changes in serum androstenedione, testosterone, progesterone or 17-hydroxyprogesterone in any male cohort analysed compared to WT BL6 controls. (E-F) No changes in serum Androstenedione, testosterone or progesterone in any female cohort analysed compared to WT BL6 controls. There is no 17- hydroxyprogesterone detected in any female sample analysed.

**Supplementary Table 1.** (A) LC-MS/MS limits of quantification (LOQ) and range of calibration curves were based on 10 standards prepared as for human serum analysis (44). (B) LC-MS/MS validation: Inter day control materials for this study were prepared in two pools of mouse serum (serum #72 and #73) spiked in low (Q Low) and high (Q High) levels. Results are mean (n=3) of control materials analysed in three batches.
